# Immortalization of chicken preadipocytes by retroviral transduction of chicken TERT and TR

**DOI:** 10.1371/journal.pone.0177348

**Published:** 2017-05-09

**Authors:** Wei Wang, Tianmu Zhang, Chunyan Wu, Shanshan Wang, Yuxiang Wang, Hui Li, Ning Wang

**Affiliations:** 1 Key Laboratory of Chicken Genetics and Breeding, Ministry of Agriculture, Harbin, China; 2 Key Laboratory of Animal Genetics, Breeding and Reproduction, Education Department of Heilongjiang Province, Harbin, China; 3 College of Animal Science and Technology, Northeast Agricultural University, Harbin, China; University of Nebraska Medical Center, UNITED STATES

## Abstract

The chicken is an important agricultural animal and model for developmental biology, immunology and virology. Excess fat accumulation continues to be a serious problem for the chicken industry. However, chicken adipogenesis and obesity have not been well investigated, because no chicken preadipocyte cell lines have been generated thus far. Here, we successfully generated two immortalized chicken preadipocyte cell lines through transduction of either chicken telomerase reverse transcriptase (chTERT) alone or in combination with chicken telomerase RNA (chTR). Both of these cell lines have survived >100 population doublings *in vitro*, display high telomerase activity and have no sign of replicative senescence. Similar to primary chicken preadipocytes, these two cell lines display a fibroblast-like morphology, retain the capacity to differentiate into adipocytes, and do not display any signs of malignant transformation. Isoenzyme analysis and PCR-based analysis confirmed that these two cell lines are of chicken origin and are free from inter-species contamination. To our knowledge, this is the first report demonstrating the generation of immortal chicken cells by introduction of chTERT and chTR. Our established chicken preadipocyte cell lines show great promise as an *in vitro* model for the investigation of chicken adipogenesis, lipid metabolism, and obesity and its related diseases, and our results also provide clues for immortalizing other avian cell types.

## Introduction

The chicken is an important agricultural animal throughout the world and is a significant non-mammalian vertebrate model for developmental biology, virology and immunology. In addition, chickens show high glycemia and low sensitivity to exogenous insulin (particularly in adipose tissues), making them a potential model for studies on human obesity, insulin resistance and type 2 diabetes [1–5]. In the broiler chicken industry, excessive fat deposition has been a growing concern that urgently needs to be addressed, because it not only reduces carcass yield and feed efficiency but also causes processing difficulties and environmental pollution.

The established immortal preadipocyte cell lines are indispensable for studying adipocyte differentiation [6]. Most of our knowledge of adipocyte differentiation has been derived from experiments using immortal mammalian preadipocyte cell lines. Of these cell lines, the mouse 3T3-L1 preadipocyte line has been widely used to study adipocyte differentiation [6]. Accumulating studies indicate that there are some clear differences in adipocyte differentiation and lipogenesis between mammals and birds [7–10], suggesting that our current knowledge of adipogenesis may not apply to chicken adipogenesis. Therefore, to gain a deeper understanding of chicken adipogenesis and excessive fat deposition, it is essential to generate immortal chicken preadipocyte cell lines. Unfortunately, no immortal chicken preadipocyte cell lines are available to date.

Generally, chicken cells rarely immortalize spontaneously because of their low spontaneous mutation rate [11]. Oncogenic viruses and viral oncogenes can be used to immortalize chicken cells. For example, Marek’s Disease Virus (MDV) and Avian Leukosis Virus can be used to immortalize several specific avian cell types [12,13]. However, because viruses are host- and cell type-specific, oncogenic viruses cannot be widely used in chicken cell immortalization. Viral oncogenes, such as the SV40 Large-T antigen, adenovirus E1A and E1B, papilloma virus E6 and E7, CELO virus orf22 and GAM-1 [14–16], have been used to immortalize avian cells. The main drawback of this approach is that the generated cell lines often lose cell cycle and apoptosis control due to the inhibition of the pRB and p53 pathways, respectively, which ultimately results in malignant cell transformation [17,18].

Telomerase activity restoration is an ideal method to immortalize mammalian cells [19–21]. Telomeres play an essential role in maintaining chromosome stability and determining cellular life span. Telomerase is a ribonucleoprotein complex that extends and maintains telomeres. The telomerase enzyme complex has two major subunits contributing to enzymatic activity—a catalytic subunit with reverse transcriptase activity (TERT) [22,23] and a structural RNA component (TR) that serves as a template for TERT to add hexameric repeats to the telomere terminal [24]. Telomerase activation is required for cells to overcome replicative senescence and become immortal [25,26]. For most human and other mammalian cell types, human TERT (hTERT) is the rate-limiting component of telomerase [19,27]. Transfection with hTERT alone can extend cellular life span and immortalize a number of cell types without malignantly-transformed phenotypes [19,20,28,29]. To date, hTERT has been widely used for human and many other mammalian cell immortalizations.

Several previous studies have attempted to immortalize chicken cells using hTERT, but their results are controversial. Previous studies have shown that the introduction of hTERT could not restore cellular telomerase activity and immortalize telomerase-negative chicken cells, such as chicken embryo fibroblasts (CEFs) [30,31], suggesting that hTERT cannot be used to immortalize chicken cells. However, a recent study showed that ectopic expression of hTERT could immortalize chicken feather keratinocyte stem cells [32]. These controversial results may indicate that hTERT-mediated chicken cell immortalization is cell type-specific, possibly due to species differences in the expression of telomerase components. Therefore, to obtain the maximum possibility of immortalizing various chicken cell types, the ideal method may be to use chicken telomerase components instead of hTERT for telomerase activity restoration.

Chicken telomerase activity has been reconstituted *in vitro* in a rabbit reticulocyte lysate system by assembly of chTERT and chTR [33]. Correlation analysis of telomerase activity and gene expression levels of chTERT and chTR in various chicken tissues suggested that, unlike human telomerase, the rate-limiting component of chicken telomerase activity may be either or both chTERT and chTR [34], suggesting that the reconstruction of chicken telomerase activity in chicken cells requires the consideration of using either, or perhaps both, chTERT and chTR. To date, there are no reports of the generation of immortalized chicken cells through restoration of cellular telomerase activity with chicken telomerase components [35]. In the present study, we established two immortalized chicken preadipocyte cell lines by retroviral transduction of chTERT and chTR, which maintained the differentiation capacity and morphological characteristics of primary chicken preadipocytes. These two cell lines could serve as *in vitro* models for elucidating the mechanisms of chicken adipocyte differentiation and lipid metabolism and as potential models for investigating human obesity, insulin resistance and related diseases.

## Methods

### Ethics statement

This study was approved by the Laboratory Animal Management Committee of the Northeast Agricultural University, China. All animal studies were performed under the Guide for the Care and Use of Laboratory Animals of the National Institutes of Health, and under the Guidance Suggestion of Caring for Laboratory Animals of the Ministry of Science and Technology of the People’s Republic of China (document No. [2006]398). Ten-day-old Arbor Acres (AA) broiler chickens were used as experimental animals. The experimental chicks were humanely euthanized by CO_2_ asphyxiation followed by thoracotomy to ensure death, and every effort was made to minimize suffering.

### RNA extraction and cDNA synthesis

Total RNA was isolated from cells or tissue samples using an E.Z.N.A. total RNA kit II (Omega Bio-tek) according to the manufacturer’s protocol, and first-strand cDNA was synthesized from 1 μg of RNA template with oligo dT or random primers using ImProm-II reverse transcriptase (Promega).

### Cloning of chTERT and chTR

The full-length chTERT coding region was amplified by RT-PCR from AA broiler embryos (embryonic day 4) as three overlapping fragments (chTERT-T1, -T2 and -T3), using the PrimeSTAR HS DNA Polymerase (TaKaRa). The primers are shown in Table 1. ChTERT-T1 and chTERT-T2 were connected by overlap extension PCR, and the resultant fragment chTERT-T1/T2 was inserted into *Sal* I and *Nco* I sites of the pMD18T-chTERT-T3 vector to yield pMD18T-chTERT. The chTR was PCR-amplified from the genomic DNA of AA broilers with the primers listed in Table 1 and cloned into the pMD18T vector. The resultant plasmids were verified by restriction enzyme digestion and sequencing.

**Table 1:** Primers used for gene cloning and expression analysis.

Primer name	Application	Primer sequences
**chTERT-T1**	Gene cloning	Forward (F): 5'-AAGTCGACCGTGGGGCCCGCTGCACGGCAG-3'
		Reverse (R): 5'-GCTCTGACTGGATAACTGCTGGAAGCAGATGGGCCGGGG-3'
**chTERT-T2**	Gene cloning	F: 5'-TTCCAGCAGTTATCCAGTCAGAGCGAAGTCATC-3'
		R: 5'-CCATACGCAGTCATTCACTCTCATCTTCCACATC-3'
**chTERT-T3**	Gene cloning	F: 5'-GCCATAACAAATGCCGGTTCTTTAAAAACGTG-3'
		R: 5'-CGCTCGAGAGACCTTCATCCCTTAGTCCAG-3'
**chTERT-T1/T2**	Gene cloning	F: 5'-GTCGACTTGTGGGGTCCGCTGCAC-3'
		R: chTERT-T2 R
**chTR**	Gene cloning	F: 5'-ACGCGTCGACACGCGTGGCGGGTGGAAGGC-3'
		R: 5'-CCGCTCGAGGCGTGTGGGAGCGACGCCGTC-3'
**chTERT**	Semi-quantitative RT-PCR	F: 5'-ATGCCTTCATTGTCAAACTGTCC-3'
		R: 5'-GATGGTTCCGTCACCGTCTTC-3'
**chTR**	Semi-quantitative RT-PCR	F: 5'-CGCTGTGCCTAACCCTAATCG-3'
		R: 5'-CGCTCCCGTTTGCTCTGC-3'
**18S**	Semi-quantitative RT-PCR	F: 5'-TAGATAACCTCGAGCCGATCGCA-3'
		R: 5'-GACTTGCCCTCCAATGGATCCTC-3'
**A-FABP**	Quantitative real-time RT-PCR	F: 5'-AGTTTGTGGGCACCTGGAAGC-3'
		R: 5'-CCATCCACCACTTTCCTCTT-3'
**C/EBPα**	Quantitative real-time RT-PCR	F: 5'GGAGCAAGCCAACTTCTACGC-3'
		R: 5'CTCGTTCTCGCAGATGTCGC-3'
**PPARγ**	Quantitative real-time RT-PCR	F: 5'-TACATAAAGTCCTTCCCGCTGACC-3'
		R: 5'-TCCAGTGCGTTGAACTTCACAGC-3'
**PLIN**	Quantitative real-time RT-PCR	F: 5'-GGGGTGACTGGCGGTTGTA-3'
		R: 5'-GCCGTAGAGGTTGGCGTAG-3'
**FAS**	Quantitative real-time RT-PCR	F: 5'-AAGGAGGAAGTCAACGG-3'
		R: 5'-TTGATGGTGAGGAGTCG-3'
**GOS2**	Quantitative real-time RT-PCR	F: 5'-CGGGGCGAAAGAGCTGAG-3'
		R: 5'-AGCACGTACAGCTTCACCAT-3'
**GAPDH**	Quantitative real-time RT-PCR	F: 5'-AGAACATCATCCCAGCGT-3'
		R: 5'-AGCCTTCACTACCCTCTTG-3'

### Retroviral vector construction and virus production

The recombinant plasmids pLXRN-chTERT and pLPCX-chTR were constructed by inserting the chTERT and chTR into the pLXRN (neomycin) and pLPCX (puromycin) retroviral expression vectors (Clontech), respectively. The detailed procedures for virus production, concentration, and viral titration were followed according to the retroviral gene transfer and expression user manual (Clontech). Briefly, the retroviral vector and envelope vector (pVSV-G) were co-transfected into GP2-293 packaging cells using the FuGENE HD transfection reagent (Roche). At 48 to 72 h after transfection, the retrovirus-containing supernatants were harvested and filtered through a 0.45 μm filter. The supernatants were concentrated using the Retro-X Concentrator (Clontech) according to the manufacturer’s protocol. The viral titer was determined by infecting NIH 3T3 cells with serially-diluted concentrations of viral supernatants, and then a drug-resistance colony assay was performed in which antibiotic selection of the NIH 3T3 cells gives rise to a countable number of colonies after approximately 10 to 14 days. The viral titer was greater than 1×10^8^ colony forming units/ml.

### Isolation of chicken preadipocytes and cell culture

Primary chicken preadipocytes (PCPs) were isolated from the abdominal adipose tissue of 10-day-old AA broiler chickens as previously described [36]. Briefly, chicken adipose tissue was collected and washed with PBS, and visible blood vessels were removed. The adipose tissue was minced into sections of approximately 1 mm^2^ with scissors and incubated with 10 ml of digestion buffer (DMEM/F12, 100 mM HEPES, 1.5% BSA, pH 7.4) supplemented with 2 mg/ml collagenase (Type 1, Invitrogen) with shaking for 65 min at 37°C. Flask contents were mixed and filtrated through nylon screens with 100- and 25-μm mesh openings to remove undigested tissue and large cell aggregates. The filtered cells were centrifuged at 300 x g for 10 min to separate the floating adipocytes from the pellet of stromal-vascular cells. The stromal-vascular cells were then resuspended with erythrocyte lysis buffer, incubated at room temperature for 10 min and then centrifuged at 300 x g for 10 min. The stromal-vascular cells (including the preadipocytes) were washed with DMEM/F12 and seeded at a density of 5×10^4^ cells/ml in DMEM/F12 media supplemented with 10% FBS plus 100 units/ml penicillin and 100 μg/ml streptomycin, at 37°C with 5% CO_2_. The PCPs were serially subcultured at a 1:2 split ratio until senescence. The following formula was used to determine the population doublings of each subculture and to establish the PD growth curve: PD = ln(N_finish_/N_start_)/ln2, where PD is the number of population doublings, ln is the natural logarithm, N_start_ is the number of cells initially seeded, and N_finish_ is the total number of cells recovered at subculture.

GP2-293 cells, NIH 3T3 cells, DF-1 cells, and HEK-293 cells were grown in DMEM supplemented with 10% FBS plus 100 units/ml penicillin and 100 μg/ml streptomycin, at 37°C with 5% CO_2_.

### Retroviral transfection and screening of immortalized chicken preadipocytes

The PCPs were plated on five 60-mm dishes at a density of 2 × 10^5^ cells per dish, and the cells were individually infected with the five recombinant retroviruses chTERT, chTR, chTERT/chTR, empty pLXRN and pLPCX, followed by selection with the designated antibiotic at the optimized concentration for two weeks. For combination infections, the PCPs were first infected with chTERT retroviruses and selected with G418 for two weeks, and then infected with chTR retroviruses and selected with puromycin for two more weeks. The detailed procedures of retroviral transfection and screening were followed according to the retroviral gene transfer and expression user manual (Clontech). After retrovirus infection and drug selection, all cells were continuously subcultured at a 1:2 split ratio. When ICP1 and ICP2 reached PD 36 and PD 20, respectively, their split ratio was increased to 1:4.

### β-gal assay

The β-gal assay was performed using the senescence β-galactosidase staining kit (Beyotime) according to the manufacturer’s protocol. Briefly, the cells were washed with PBS and fixed in 4% paraformaldehyde for 15 min at room temperature. Next, the cells were washed three times with PBS and stained with X-gal solution for 12 h at 37°C (no CO_2_). The percentage of β-gal positive cells was determined by counting 500 cells per sample, and photographs were taken using a phase-contrast microscope (Leica).

### Semi-quantitative and quantitative real-time RT-PCR gene expression analysis

The expression levels of chTERT and chTR mRNA were analyzed by semi-quantitative RT-PCR. The 18S rRNA gene served as an internal standard, and the semi-quantitative PCR primers are shown in Table 1. The PCR products were analyzed by electrophoresis on 2% agarose gels.

The expression levels of adipocyte differentiation marker genes (*PPARγ*, *C/EBPα*, *FAS*, *GOS2*, *PLIN* and *A-FABP*) were analyzed by quantitative real-time RT-PCR with SYBR Green PCR Master Mix (Roche). The primers are listed in Table 1. PCR was carried out in an ABI 7500 real time PCR system (Applied Biosystems). PCR results were recorded as threshold cycle numbers (Ct) (the original data are given in S1 Table). The fold change in the target gene expression, normalized to the expression of internal control gene (GAPDH) and relative to the expression at time point 0, was calculated using the 2 ^−ΔΔCT^ method as previously described [37]. The results are presented as the mean ± SD of three independent experiments.

### Telomeric repeat amplification protocol (TRAP) assay

TRAP assays were performed using the TRAPEZE RT Telomerase Detection Kit (Millipore) according to the manufacturer’s protocol. This assay quantifies telomerase activity by measuring real-time fluorescence emission using quantitative PCR. Briefly, cells were lysed in 200 μl of CHAPS buffer. Aliquots of cell lysates (1 μg of protein/well) were assayed in a 96-well quantitative PCR plate. A standard curve was generated by 1:10 serial dilutions of TSR8 (S2 Table). Telomerase-positive cell extract (prepared from 1 × 10^3^ cells provided in the kit) was used as a telomerase-positive control. Heat-inactivated samples (HT), minus-telomerase control (MTC) and no template control (NTC) samples were used as telomerase-negative controls. Telomerase activity (total product generated) was calculated by comparing the average Ct values from the sample wells against the standard curve generated by the TSR8 control template (the original data are given in S2 Table).

### DNA ploidy analysis

DNA ploidy analysis was performed as previously described [38,39]. Briefly, cells were fixed and stained with a saturating concentration (40 μg/ml) of propidium iodide and 100 μg/ml of RNase. Two aliquots of each sample were stained and mixed in a 1:1 ratio with normal chicken cells (PCPs and CEFs) that were processed identically and served as an internal standard. Flow cytometric ploidy analysis was performed with a FACScan flow cytometer (Beckman Coulter) and analyzed using ModFit LT software. For each sample, 1 × 10^4^ to 2 × 10^4^ cells were analyzed. The DNA content was expressed as a DNA index (DI), and the DI was obtained by dividing the modal fluorescence channel of the G0/G1 peak of the abnormal cells by the modal fluorescence channel of the residual G0/G1 normal cells present in the sample [38].

### Anchorage independence of growth

The cells were harvested and suspended in 0.3% Noble agar in DMEM/F12 medium containing 10% FBS at a density of 800 cells/ml. A total of 4 × 10^3^ ICP1 at PD 100 or ICP2 at PD 100 were seeded onto 10-cm dishes by pouring 5 ml of cells in 0.3% Noble agar on top of 0.6% Noble agar in DMEM/F12 containing 10% FBS. A total of 1 × 10^3^ HEK 293 cells served as a positive control. Colonies larger than a diameter of 0.125 mm were counted by phase contrast microscopy (Leica) after incubation for 3 weeks. All experiments were performed three times in triplicate.

### Induction of chicken preadipocyte differentiation

Chicken preadipocytes were plated in 6-well plates at a density of 2–3 × 10^5^ cells per well and cultured with DMEM/F12 media containing 10% FBS and 160 μM sodium oleate (Sigma). The medium was changed every day until day 5 of culture. Adipocyte differentiation was examined by microscopy and Oil Red O staining.

### Oil Red O staining and its quantification

Cells were washed with PBS, and fixed with 10% formalin in PBS for 1 h at room temperature. After fixation, cells were washed with ddH_2_O and stained with Oil Red O working solution (Oil Red O dye in 60% isopropanol) at room temperature for 10 min. Cells were then washed immediately with ddH_2_O and analyzed under a microscope (Leica). Quantitative assessment was obtained by spectrophotometric analysis of the absorbance of the extracted dye at 500 nm (the original data are given in S3 Table).

### Isoenzyme analysis

An isoenzyme analysis of lactate dehydrogenase (LDH) and nucleoside phosphorylase (NP) was carried out using the AuthentiKit (Innovative Chemistry) gel electrophoresis system following the manufacturer’s instructions and the standard protocol [40]. Briefly, 1×10^7^ cells were harvested from a T-75 flask by trypsinization and centrifugation. The cell pellet was approximately 100 μL. The pellet was resuspended in an equal volume of Cell Extraction Buffer (100 μL) and allowed to stand on ice for 15 to 30 min. After centrifugation to remove the cell debris, approximately 100 μl of the supernatant was transferred to a new tube, and 100 μl of Enzyme Stabilizer was added and mixed. The enzyme activity of the cell extract was quantified with the Quench-A-Zyme Reagent by spectrophotometry according to the manufacturer’s protocol. Human HeLa cells and murine L929 cells were used as standard references. Chicken DF-1 cells and chicken muscle tissues were used as positive controls. The profiles of LDH and NP isoenzymes were evaluated by agarose electrophoresis. The origin of the cell lines was determined by comparing the migration distances of the analyzed isoenzymes in the samples, controls and standard references.

### PCR identity testing

11 species-specific primer pairs for human, Syrian hamster, mouse, rat, dog, African green monkey, rabbit, Chinese hamster, pig, cow and chicken were synthesized based on published literature [41–43], and the primers are listed in Table 2. Genomic DNA was extracted from cultured cells derived from these 11 different species. These cell lines included A549, A9, BHK21, RIN-m5F, MDCK, VERO, RK13, CHO, PK15, MDBK and DF-1 cells. All PCR analyses were carried out as described in the published literature [41–43].

**Table 2:** Species-specific primers used in this study.

Cell Line	Species	Gene	NCBI accession number	Sequence	Size (bp)	References
**A549**	human	β-globin	M34058	F: 5'-caagacaggtttaaggagacca-3'	1411	[41]
				R: 5'-gcagaatccagatgctcaagg-3'		
**A9**	mouse	cox I	J01420	F: 5'-ATTACAGCCGTACTGCTCCTAT-3'	150	[42]
				R: 5'-CCCAAAGAATCAGAACAGATGC-3'		
**BHK21**	Syrian hamster	β-globin		F: 5'-AGGTGATCCACTCCTTCGCT-3'	~1200	[41]
				R: 5'-TGTTCTCTAGGGAACAAGTGACTT-3'		
**RIN-m5F**	rat	cox I	NC001665	F: 5'-CGGCCACCCAGAAGTGTACATC-3'	196	[42]
				R: 5'-GGCTCGGGTGTCTACATCTAGG-3'		
**MDCK**	dog	cox I	U96639	F: 5'-GAACTAGGTCAGCCCGGTACTT-3'	153	[42]
				R: 5'-CGGAGCACCAATTATTAACGGC-3'		
**VERO**	African green monkey	cox I	AF312703	F: 5'-CCTCTTTCCTGCTGCTAATG-3'	222	[42]
				R: 5'-TTTGATACTGGGATATGGCG-3'		
**RK13**	rabbit	cox I	NC001913	F: 5'-CGGGAACTGGCTTGTCCCCCTG-3'	151	[42]
				R: 5'-AACAGTTCAGCCAGTCCCCGCC-3'		
**CHO**	Chinese hamster	cytochrome b	AB033693	F: 5'-GTGACCCATATCTGCCGAGAT-3'	293	[42]
				R: 5'-CATTCTACTAGGGTGGTGCCC-3'		
**PK15**	pig	calpastatin	EU137105.1	F: 5'-ACTGCCAGCAGCCTAAATGTAT-3'	517	UniSTS:516113
				R: 5'-TCCCTAACTTGCCAGTCTTAGC-3'		
**MDBK**	cow	BT225		F: 5'-TCACTGGCTTACAACTAGGG-3'	272	UniSTS:64730
				R: 5'-TGGAGATGAGTTTGACTAAG-3'		
**DF-1**	chicken	mitochondrial DNA	AB086102	First primer	197	[43]
				F:5'-TAGTAGAGTGAGCCTGAGGGGGAT-3'		
				R: 5'-CGATGTTGGATCAGGACAAC-3'		
				Second primer		
				F: 5'-GTATTCCCGTGCAAAAACGAG-3'		
				R: 5'-CTTAGTGAAGAGTTGTGGTCTG-3'		

### Statistical analysis

The data are presented as the mean ± SD; the Student’s t-test and Duncan’s multiple range test were used to assess the difference between individual groups, and *P* ≤ 0.05 was considered statistically significant.

## Results

### Cloning of chTERT and chTR

Two core components of chicken telomerase, chTERT and chTR, were cloned as described in the *Methods*. The full-length coding sequence of chTERT was 4097 bp long, and encoded a protein of 1346 amino acids (aa). Its aa sequence was identical to the *Gallus gallus* TERT sequence [GenBank: NP_001026178], except for one transition mutation (L-F mutation at aa 484). The chTR was 464 bp long, contained an 11-nucleotide template sequence (5'-CUAACCCUAAU-3'), and had one deletion mutation at nt 227 when compared with the *Gallus gallus* telomerase RNA sequence [GenBank: NR_001594].

### Overexpression of chTERT either alone or in combination with chTR can immortalize chicken preadipocytes

**Fig 1:**
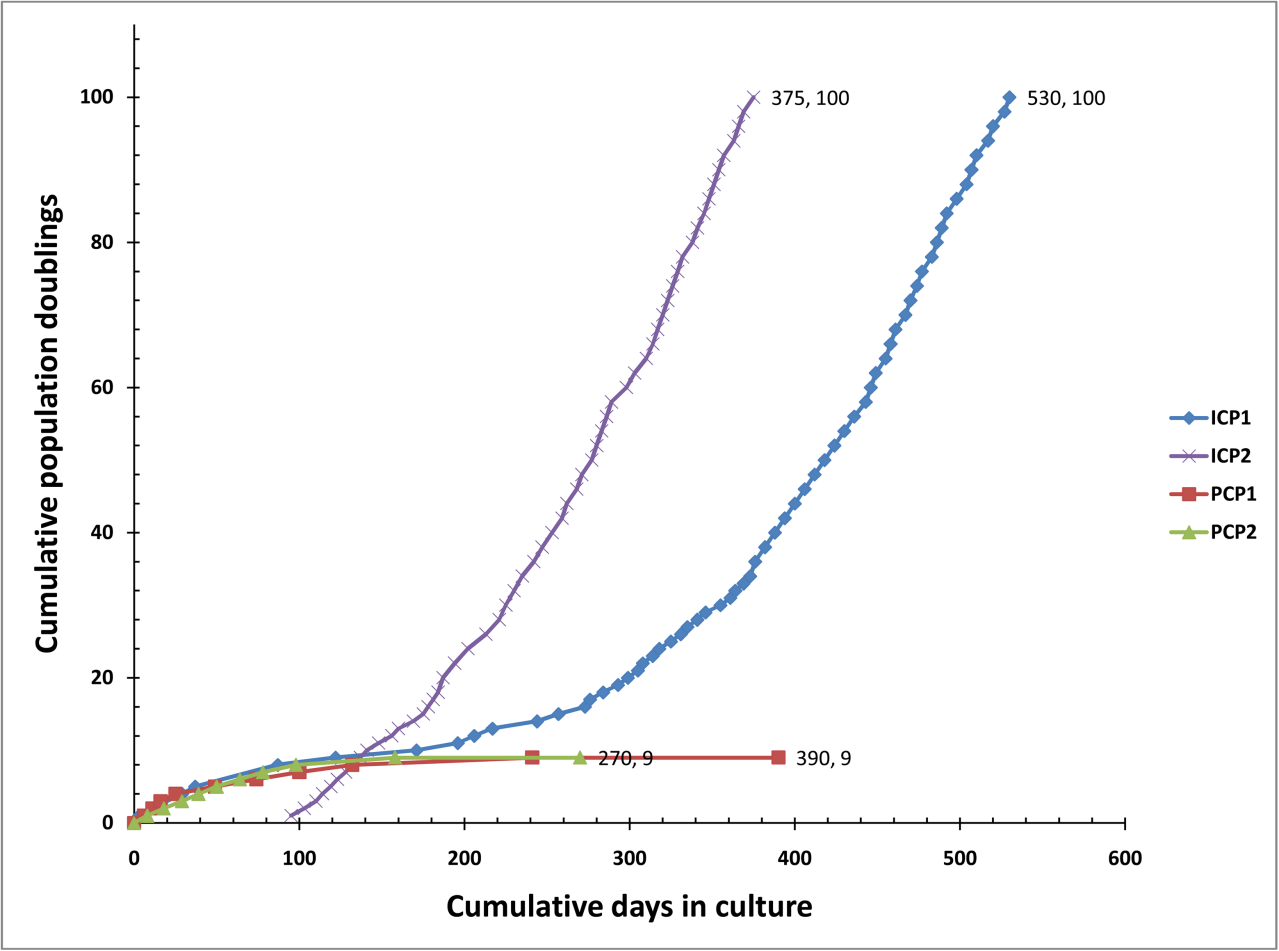
Cumulative population doublings of immortalized and primary chicken preadipocytes. Immortalized chicken preadipocytes (ICP1, ICP2) and two batches of primary chicken preadipocytes (PCP1, PCP2) were serially subcultured for more than 1 year. Both ICP1 and ICP2 cell lines grew beyond PD 100. The PCP1 and PCP2 cells were no longer capable of reaching confluence by PD 9. The cumulative PDs of ICP2 were calculated from 95 days (after two rounds of retrovirus infection and drug selection).

To determine whether chTERT and chTR can immortalize chicken preadipocytes, we prepared recombinant retroviruses encoding chTERT (pLXRN-chTERT) and chTR (pLPCX-chTR), and their respective empty vector retroviruses (pLXRN and pLPCX) as controls (mock transduction). PCPs were isolated from the abdominal adipose tissues of 10-day-old Arbor Acres (AA) broiler chickens and infected individually with the four recombinant retroviruses (chTERT, chTR, pLXRN and pLPCX) and a combination of chTERT and chTR retroviruses. After retroviral infection and drug selection, the resulting cells were continuously subcultured at a 1:2 split ratio when confluent. In parallel, to determine the maximum *in vitro* life span of PCPs, we continuously subcultured two batches of PCPs (PCP1 and PCP2) at a 1:2 split ratio for more than 1 year. The results showed that with increasing population doublings (PDs), PCP1 and PCP2 had slower growth rates and took longer to achieve confluence (Fig 1). At PD 9, PCP1 and PCP2 were no longer capable of reaching confluence and were maintained in a quiescent state for 258 and 172 days, respectively (Fig 1). Similar to PCPs, preadipocytes infected with chTR or two empty vector retroviruses (pLXRN and pLPCX), failed to reach confluence between PDs 8 to 10. Only the chTERT retrovirus-infected preadipocytes and the chTERT/chTR-coinfected preadipocytes eventually bypassed the replicative senescence and grew beyond 100 PDs (Fig 1). For the first 171 days (the first 10 PDs), the chTERT retrovirus-infected preadipocytes proliferated similarly to primary cells and showed growth inhibition during PDs 5 to 10; subsequently, a small group of the selected cells gained a proliferative advantage and grew continuously afterwards (Fig 1). In contrast to the chTERT retrovirus-infected preadipocytes, the chTERT/chTR-coinfected preadipocytes appeared to avoid replicative senescence and proliferated consistently and at a high level during serial culture (Fig 1). These two cell lines were continuously passaged at a 1:2 or 1:4 split ratio for more than a year, and their cumulative PDs were over 100 (Fig 1), which far exceeded the maximum *in vitro* life span of PCPs (PDs 8 to 10) (Fig 1). According to the definition of immortal cell lines [40], both cell lines can be considered as immortal. We designated the selected chTERT retrovirus-infected preadipocytes as immortalized chicken preadipocyte 1 (ICP1), and the selected chTERT/chTR-coinfected preadipocytes as immortalized chicken preadipocyte 2 (ICP2).

### ICP1 and ICP2 retain the morphological features of PCPs and escape cellular senescence

**Fig 2:**
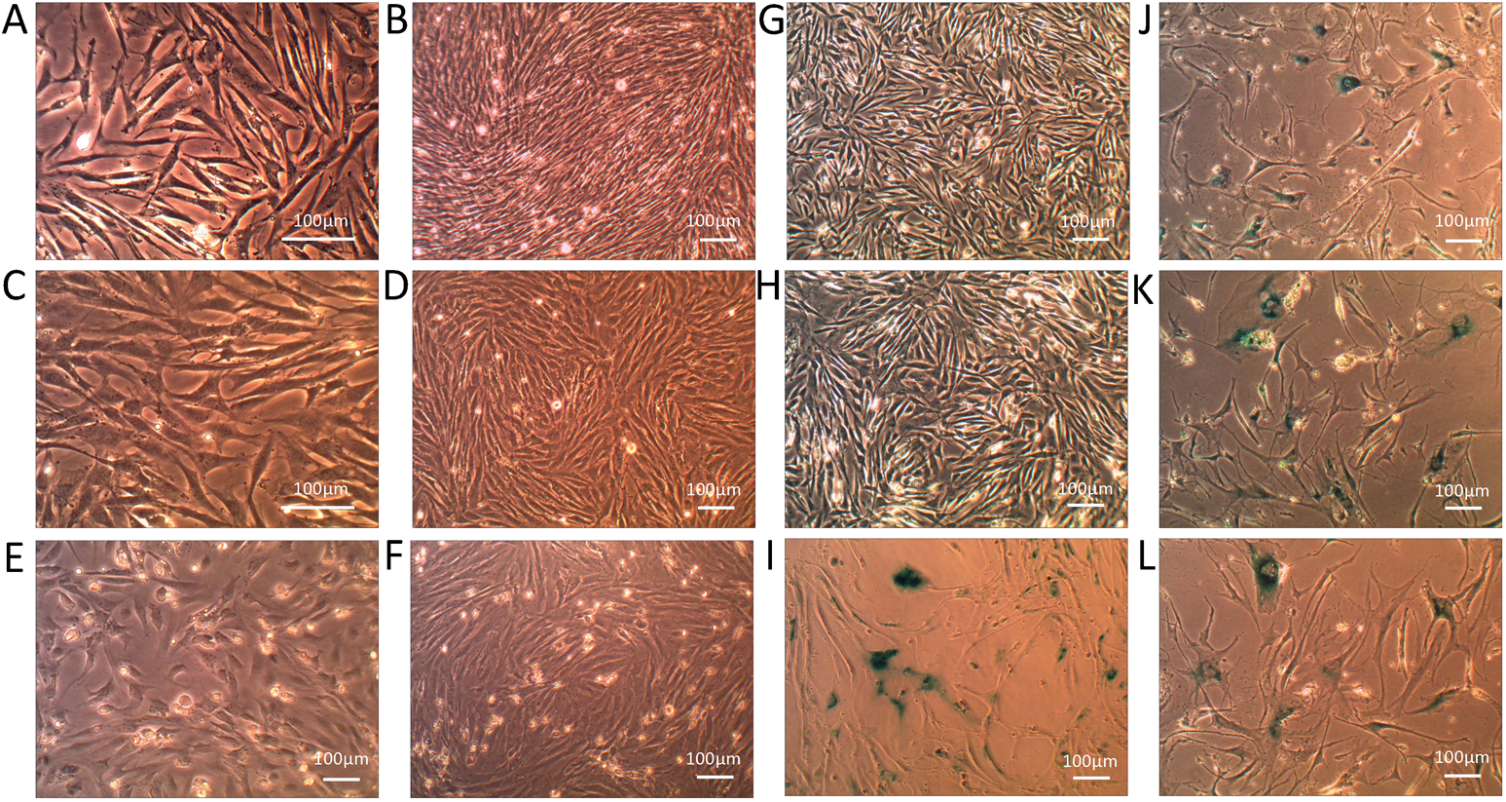
Cell morphology comparisons and β-gal staining of immortalized and primary chicken preadipocytes. (A) and (B) Light microscopy of ICP1 cells at partial and full confluence at PD 22; (C) and (D) Light microscopy of ICP2 cells at partial and full confluence at PD 15; (E) Light microscopy of the PCPs at partial confluence 1 day after culture; (F) Light microscopy of the PCPs at confluence at PD 2; (G) β-gal staining of ICP1 at PD 100; (H) β-gal staining of ICP2 at PD 100; (I) β-gal staining of the senescent PCPs at PD 9; (J) β-gal staining of chTR retrovirus-infected chicken preadipocytes at PD 10; (K) β-gal staining of empty vector (pLXRN) retrovirus-infected chicken preadipocytes at PD 8; (L) β-gal staining of empty vector (pLPCX) retrovirus-infected chicken preadipocytes at PD 8. Scale bar, 100 μm.

Morphologically, ICP1 at PD 22 (Fig 2A and 2B) and PD 100 (Fig 2G), and ICP2 at PD 15 (Fig 2C and 2D) and PD 100 (Fig 2H) grew as adherent cells in culture and assumed multipolar or bipolar fibroblast-like shapes that were similar to the morphology of PCPs (Fig 2E and 2F). No obvious cellular morphological differences were observed in the two cell lines at early and late population doubling levels (Fig 2A, 2B, 2C, 2D, 2G and 2H). At subconfluence, ICP1, ICP2 and PCPs were widely spread out on the culture surface and randomly oriented, with extended pseudopodia towards other cells (Fig 2A, 2C and 2E); however, at confluence they were bipolar and less spread out (Fig 2B, 2D and 2F). Like PCPs, ICP1 and ICP2 grew adherently as a monolayer when at high cell density, suggesting ICP1 and ICP2 remained sensitive to contact inhibition and density limitation of growth (Fig 2B, 2D and 2F). These data indicate that ICP1 and ICP2 maintained the morphological features of PCPs. In comparison, ICP1 cells were slightly smaller than PCPs, and occasionally, several lipid droplets were observed in ICP1 cells, suggesting that some ICP1 cells could spontaneously differentiate into adipocytes under normal culture conditions. In contrast, ICP2 cells were slightly bigger than PCPs and had no lipid droplets under normal culture conditions.

A morphologic examination showed that the PCPs at PD 9 (Fig 2I) and the chTR (Fig 2J), pLXRN (Fig 2K) and pLPCX (Fig 2L) retrovirus-infected preadipocytes at PDs 8 to 10 became flattened with large nuclei and spindling, which are characteristic features of senescence. Furthermore, we detected cellular senescence in ICP1, ICP2 and control cells using the β-gal assay. Consistent with the above cell growth and morphological results, the results showed that both ICP1 and ICP2 cells at PD 100 had few β-gal positive cells (Fig 2G and 2H). In contrast, the PCPs at PD 9 (Fig 2I), and the preadipocytes infected with the chTR (Fig 2J), pLXRN (Fig 2K) and pLPCX (Fig 2L) retroviruses at PDs 8 to 10 gave rise to 52% to 81% β-gal positive cells, indicating that these cells underwent cellular senescence.

### Telomerase activity is restored in ICP1 and ICP2 cell lines

**Fig 3:**
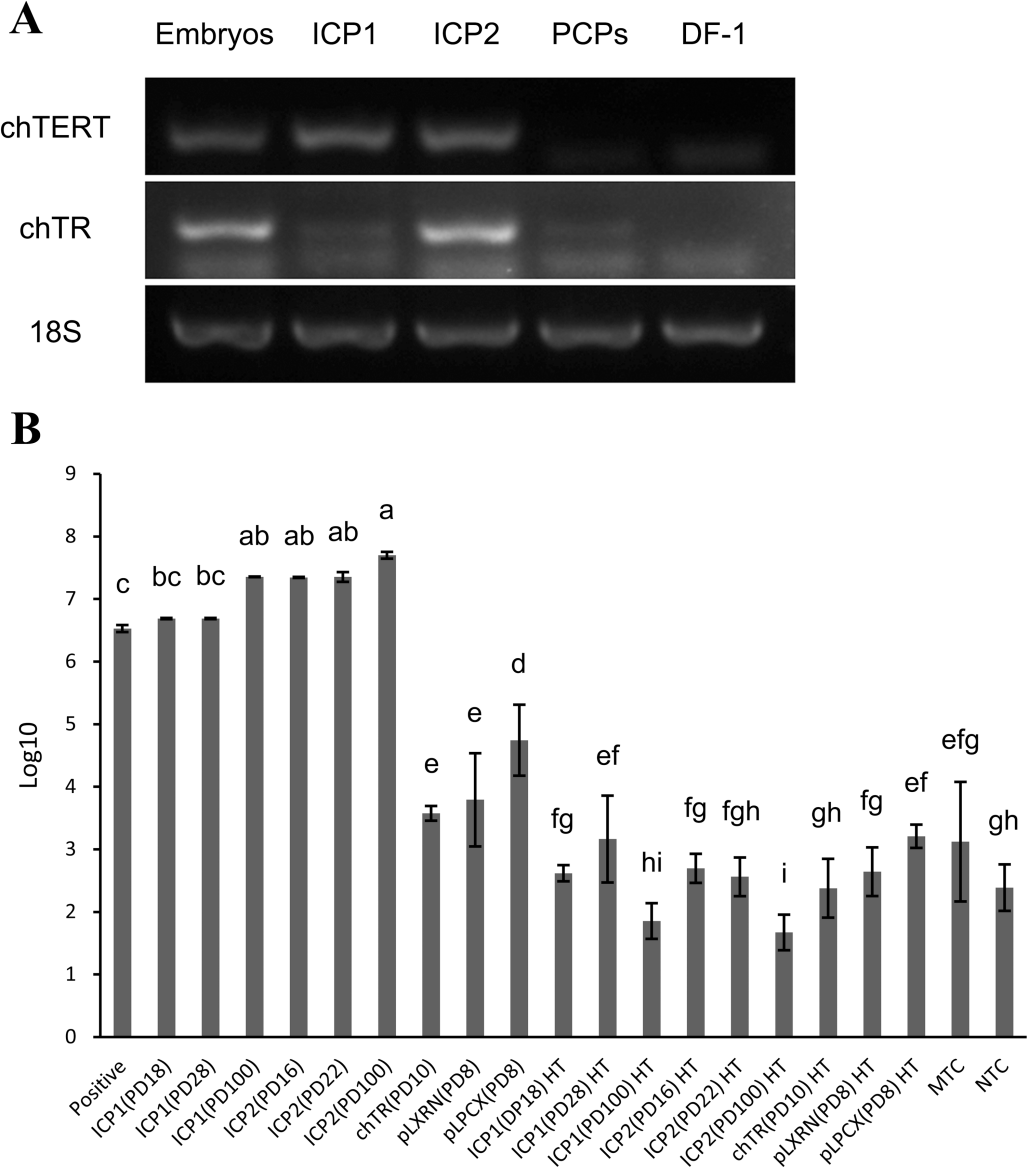
Analysis of telomerase gene expression and telomerase activity in immortalized chicken preadipocytes. (A) RT-PCR expression analyses of chTERT and chTR genes in chicken embryos, ICP1, ICP2, PCPs and DF-1 cells. The 4-day-old AA broiler embryo sample was used as a positive control and DF-1 cells as a negative control. (B) Analysis of telomerase activity in ICP1 and ICP2 cells via TRAP assay. A total of 1 × 10^3^ telomerase-positive cells were used as a telomerase-positive control. Heat-inactivated samples (HT), minus telomerase control (2 μl CHAPS lysis buffer substituted for the cell extract, MTC) and no template control (2 μl of nuclease free water substituted for the cell extract, NTC) samples were used as telomerase-negative controls. Statistical significance of each test group was evaluated by the Duncan’s multiple test (*P*<0.05).

Telomerase activity is required for cellular immortalization [25,26]. To verify whether the telomerase activity was restored in ICP1 and ICP2, we first analyzed the gene expression of chTERT and chTR in ICP1 (at PD 100), ICP2 (at PD 100) and PCPs using RT-PCR. The 4-day-old embryos of AA broilers and DF-1 cells (a non-transformed, immortalized, telomerase-negative chicken embryo fibroblast cell line) were used as positive and negative controls, respectively. Consistent with published studies [34,44], the 4-day-old embryos exhibited high expression of both chTERT and chTR, and DF-1 cells exhibited very low expression or no expression of chTERT and chTR (Fig 3A). The PCPs showed no expression of chTERT but low expression of chTR, which was similar to observations in telomerase-negative CEFs [44]. As expected, abundant chTERT mRNA was detected in both ICP1 and ICP2 cell lines, and abundant chTR was detected only in the ICP2 cell line (Fig 3A). In addition, low endogenous expression of chTR was detected in both ICP1 and PCPs (Fig 3A). Taken together, these data suggest that the exogenous chTERT and chTR genes were successfully introduced and expressed in ICP1 and ICP2 cells.

We then tested whether telomerase activity was restored in these two cell lines using a TRAP assay. As shown in Fig 3B, similar to the telomerase-positive controls, ICP1 cells (at PDs 18, 28 and 100) and ICP2 cells (at PDs 16, 22 and 100) had high telomerase activity when compared with the chTR retrovirus-infected preadipocytes (at PD 10) and empty vector retrovirus (pLXRN and pLPCX)-infected preadipocytes (at PD 8). As expected, heat treatment (HT) caused a loss of telomerase activity in the ICP1 and ICP2 samples (Fig 3B). These results indicated that these two cell lines gained and maintained telomerase activity and that chTERT, not chTR, was the rate-limiting factor for chicken preadipocyte immortalization.

### ICP1 and ICP2 are not malignantly transformed

**Fig 4:**
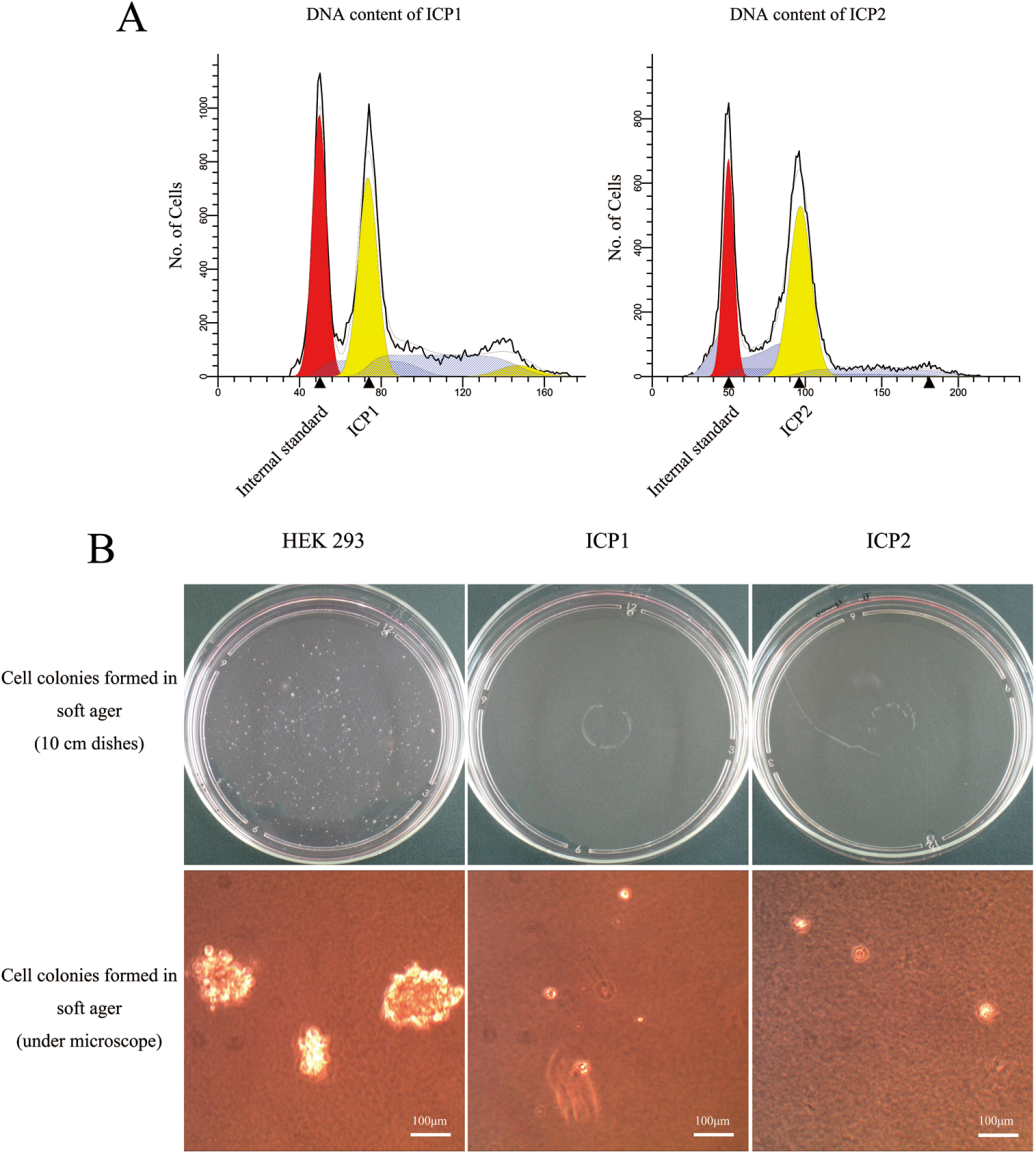
Flow cytometric ploidy analysis and soft agar colony formation assay of the immortalized chicken preadipocytes. (A) DNA content analysis of ICP1 and ICP2 cells by flow cytometry. PCPs and embryo fibroblasts of AA broiler chickens served as internal standards. (B) Soft agar colony formation assay of ICP1 and ICP2 cells. Cell colonies formed on the dishes were observed by the naked eye, and examined under a microscope. Scale bar, 100 μm.

All our results indicated that ICP1 and ICP2 cells were immortalized, which raised a question regarding whether these two cell lines were malignantly transformed. To address this question, we performed flow cytometric ploidy analysis on these two cell lines. The results showed that the DNA index (DI) of ICP1 (at PD 100) and ICP2 (at PD 100) was 1.48 and 1.92, respectively, indicating that both ICP1 and ICP2 are aneuploid (Fig 4A).

Normal cells are typically not able to grow and form cell colonies in soft agar (anchorage-dependent growth), but tumor cells or transformed cells can grow and form cell colonies in soft agar (anchorage-independent growth). Therefore, the anchorage-independent growth assay is widely used to assess cell transformation *in vitro* [40,45]. We performed the anchorage-independent growth assay on ICP1 and ICP2 cells. Adenovirus-transformed HEK 293 cells were used as the positive control, which are widely used in cell anchorage-independent growth assays [46,47]. The macro and micro examination results showed that, as expected, HEK 293 cells formed distinct cell colonies in soft agar after 3 weeks of culture (Fig 4B), and the mean colony number was 458.7 in three 10-cm dishes. In contrast, both ICP1 and ICP2 cells at PD 100 formed no clear cell colonies (only single individual cells in soft agar) (Fig 4B). Taken together, these data indicate that ICP1 and ICP2 are not malignantly transformed, although the ICP1 and ICP2 cells are aneuploid.

### ICP1 and ICP2 retain the differentiation capacity of PCPs

**Fig 5:**
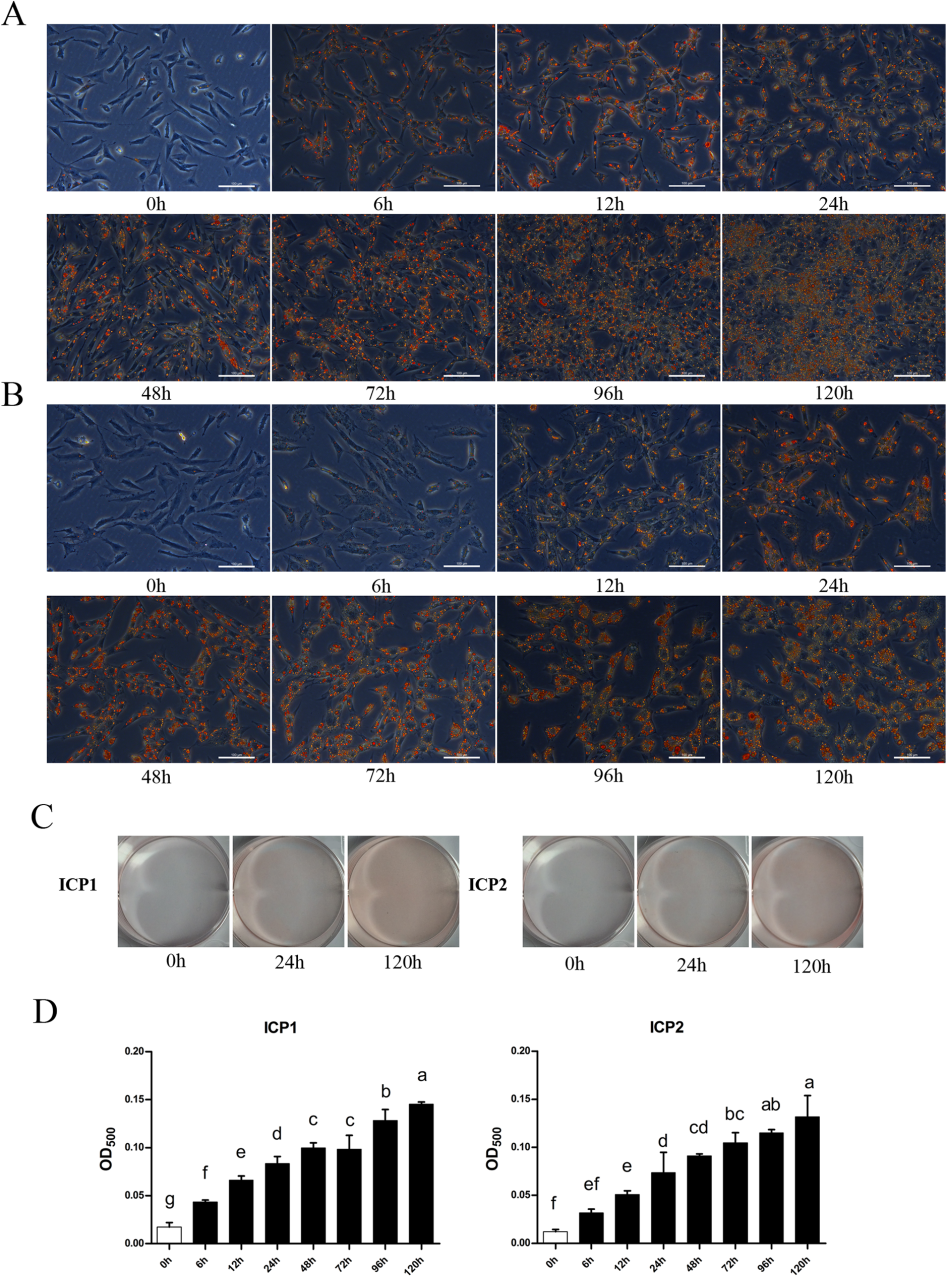
Differentiation of ICP1 and ICP2 cells induced by sodium oleate. Oil Red O-stained images of ICP1 cells at PD 100 (A) and ICP2 cells at PD 100 (B) induced with sodium oleate at 160 μM for 120 h. (C) Whole cell culture dish views of the Oil Red O staining of ICP1 and ICP2 cells induced with 160 μM sodium oleate for 120 h. (D) Quantification analysis of Oil Red O staining of ICP1 and ICP2 cells induced with 160 μM sodium oleate over time. Statistical significance of each test group was evaluated by the Duncan’s multiple test (*P*<0.05). Scale bar, 100 μm.

Next, we investigated whether ICP1 and ICP2 cells maintain differentiation capacity *in vitro*. Using the same protocol for the differentiation of PCPs [36], these two cell lines at PD 100 were induced to differentiate into adipocytes. The microscopic examination of cells stained by Oil Red O demonstrated that, similar to PCPs during differentiation into adipocytes [36], both ICP1 (Fig 5A) and ICP2 (Fig 5B) cells accumulated a large number of red perinuclear lipid droplets after sodium oleate induction. Comparatively, lipid droplets were clearly observed earlier in ICP1 cells (at 6 h) than in ICP2 cells (at 12 h), but the size of lipid droplets was larger in ICP2 cells than in ICP1 cells at 96 h and 120 h (Fig 5A and 5B). The lipid droplets accumulated in both ICP1 and ICP2 as time proceeded, as demonstrated by Oil Red O staining of whole dishes (Fig 5C). The quantification results showed that the absorbance of extracted Oil Red O dye significantly increased in ICP1 and ICP2 cells after sodium oleate induction (at 6 h), and gradually increased with time (Fig 5D).

**Fig 6:**
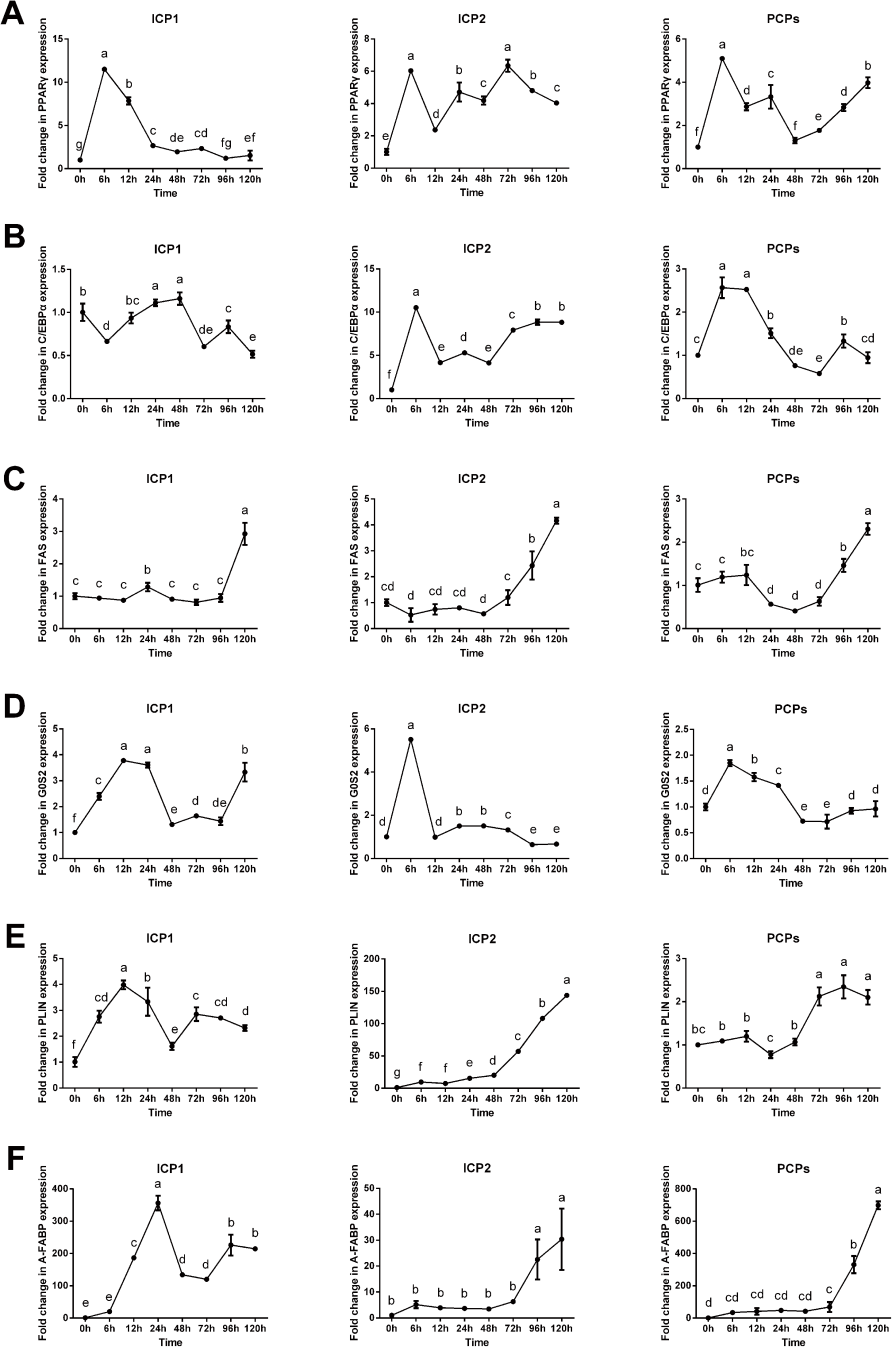
mRNA expression levels of adipocyte differentiation marker genes during the differentiation of ICP1, ICP2 and PCP cells into adipocytes. ICP1, ICP2 and PCP cells were induced to differentiate by sodium oleate, and gene expression levels of *PPARγ* (A), *C/EBPα* (B), *FAS* (C), *GOS2* (D), *PLIN* (E) and *A-FABP* (F) were analyzed by quantitative real-time RT-PCR. The results were normalized to the internal control gene (GAPDH). The mean fold changes in expression of these target genes at various time points are shown. The results are given as the mean ± SD of three independent experiments. Statistical significance was determined using the Duncan’s multiple test (P<0.05).

Furthermore, we investigated the gene expression profiles of adipocyte differentiation marker genes, including *PPARγ*, *C/EBPα*, *FAS*, *GOS2*, *PLIN*, and *A-FABP*, using quantitative real-time RT-PCR. The results showed that, on the whole, marker gene expression profiles during the differentiation in ICP1 and ICP2 cells were similar to those observed in PCPs, and the ICP2 cells tended to show more similar marker gene expression profiles to PCPs (Fig 6). As shown in Fig 6, the *PPARγ* mRNA expression in ICP1, ICP2 and PCPs significantly increased at 6 h after induction of differentiation, then decreased, and later fluctuated (Fig 6A). *C/EBPα* mRNA expression level was up-regulated at 24 h in ICP1 cells, at 6 h or 12 h in ICP2 and PCP cells, and paralleled the *PPARγ* mRNA expression pattern in the ICP2 and PCP cells (Fig 6B). *FAS* mRNA expression pattern was similar during the differentiation of ICP1, ICP2 and PCPs. *FAS* mRNA was slightly up-regulated at 12 h or 24 h and then decreased, but at late stages it began to increase significantly and reached its maximum level at 120 h (Fig 6C). *GOS2* mRNA expression was elevated at 6 h or 12 h in ICP1, ICP2 and PCP cells, and then decreased until 96 h after induction of differentiation (Fig 6D). *PLIN* mRNA expression was increased during the differentiation of ICP1, ICP2 and PCP cells, which was consistent with the formation of lipid droplets (Fig 6E). The *A-FABP* mRNA expression in ICP1 cells was rapidly increased at 12 h after the induction of differentiation and reached its maximum level at 24 h; however, in ICP2 and PCP cells, *A-FABP* mRNA expression was significantly elevated at 96 h and reached its maximum level at 120 h (Fig 6F).

Taken together, these data suggest that, similar to primary chicken preadipocytes, both ICP1 and ICP2 cell lines have the capacity to differentiate into adipocytes.

### ICP1 and ICP2 cells are of chicken origin and are free from inter-species contamination

**Fig 7:**
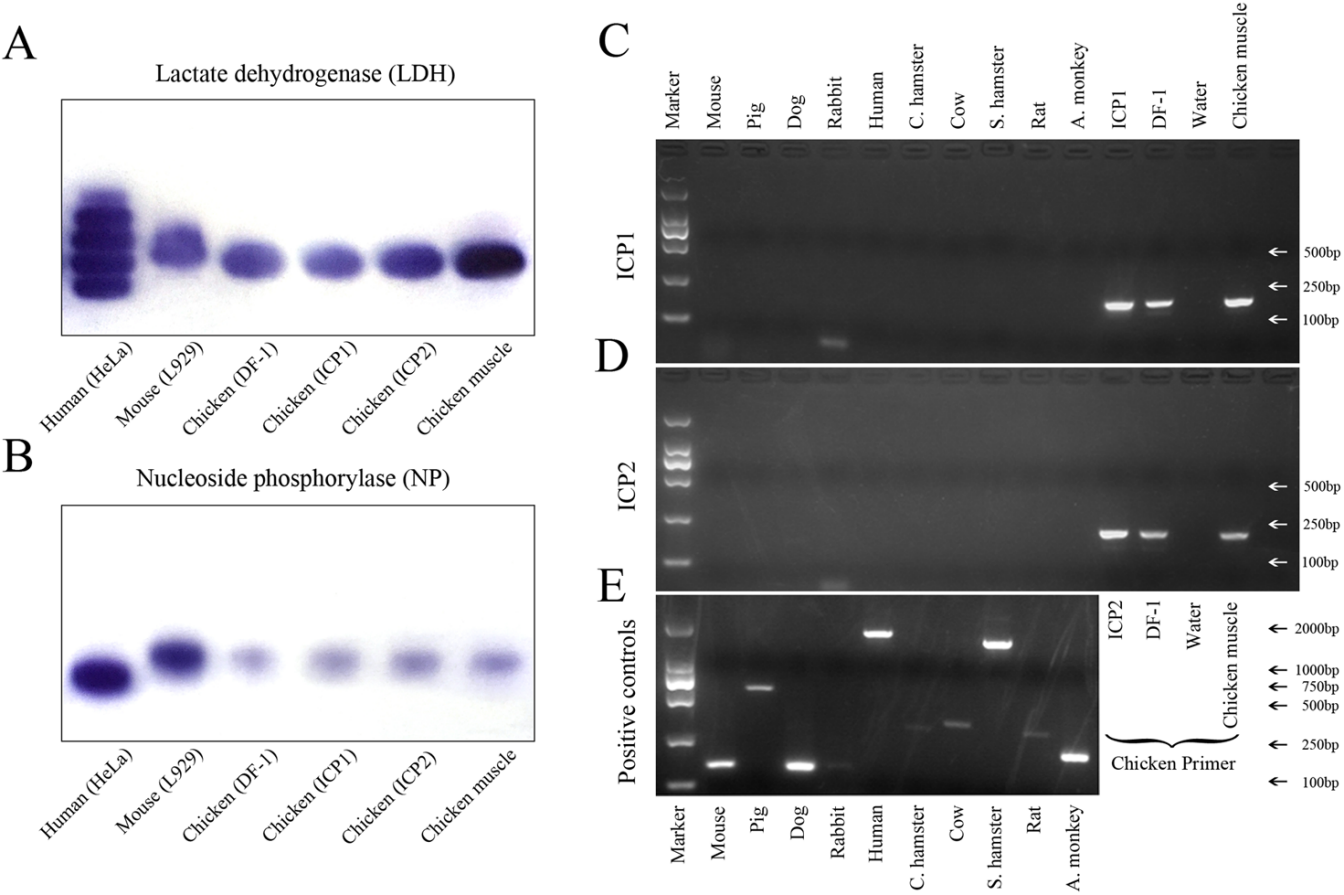
Authentication of ICP1 And ICP2 cell origin by isoenzyme analysis and PCR-based analysis. (A) and (B) Analysis of LDH and NP isoenzymes in samples from HeLa (human cells), L929 (mouse cells), DF-1 (chicken cells), ICP1, ICP2 cells and chicken muscle tissue. (C) and (D) Authentication of ICP1 and ICP2 cell lines by PCR based analysis. The same-sized PCR product was produced from ICP1, ICP2, DF-1 cells and chicken muscle tissue using chicken-specific primers, but no PCR product was produced from ICP1 and ICP2 cells using 10 other species-specific primers. (E) The amplified PCR products from the genomic DNA samples from the tested animal species.

To authenticate the origin of ICP1 and ICP2 cell lines, we performed isoenzyme analysis and PCR-based analysis on these two cell lines. The patterns of the two different isoenzymes (LDH and NP) were determined in our two cell lines. As shown in Fig 7A, LDH isoenzyme patterns were identical for ICP1, ICP2, DF-1 cells and chicken muscle tissue, but their patterns were different from those observed in human HeLa cells and mouse L929 cells. Similarly, NP isoenzyme pattern analysis showed that the pattern for ICP1, ICP2, DF-1 cells and chicken muscle tissue was identical. Their pattern was similar but not identical to that of L929 cells and clearly different from that of HeLa cells (Fig 7B). These data suggest that ICP1 and ICP2 cells are of chicken origin.

To further verify the origin of these two cell lines and exclude the possibility of contamination, we synthesized species-specific primer pairs for identifying 11 species (human, mouse, Syrian hamster, rat, dog, African green monkey, rabbit, Chinese hamster, pig, cow and chicken) based on published literature [43,48,49]. PCR analysis showed that, using these species-specific primer pairs, specific-sized PCR products were produced in the genomic DNA samples extracted from the cell lines of these species (Fig 7E), indicating that these species-specific PCR primer pairs can be used to distinguish these species. As expected, an approximately 197 bp product was detected in ICP1, ICP2, and positive controls (DF-1 cells and chicken muscle tissue) with the chicken-specific primer pair, while no amplification was detected in ICP1 and ICP2 cells with the other 10 species-specific primer pairs (Fig 7C and 7D). To verify the specificity of the PCR amplification, the chicken PCR product was validated by DNA sequencing. As expected, the sequences of PCR products from ICP1, ICP2, DF-1 cells and chicken muscle tissue were identical to the 3395–3591 bp sequence of Gallus gallus mitochondrial DNA [GenBank: AB086102].

Taken together, these data demonstrated that ICP1 and ICP2 are authentic chicken cell lines and are free from inter-species contamination.

## Discussion

In the present study, we successfully established two immortalized chicken preadipocyte lines (ICP1 and ICP2) through the introduction of chicken TERT and TR genes. These two cell lines underwent more than 100 cumulative PDs (Fig 1), which far exceeded the maximum *in vitro* life span of the primary chicken preadipocytes in our studies. They also surpassed the maximum *in vitro* life span of CEFs (approximately 20 PDs) according to published reports [50] and fulfilled the criteria defining immortalized cells [40]. Both ICP1 and ICP2 cells had high TERT expression and high cellular telomerase activity (Fig 3) and maintained the morphological features and differentiation characteristics of original chicken preadipocytes, as demonstrated by Oil Red O staining and gene expression analysis of adipocyte differentiation marker genes (Figs 2, 5 and 6). Isoenzyme analysis and PCR-based analysis confirmed that ICP1 and ICP2 cells are authentic chicken cell lines and are free from inter-species contamination (Fig 7). To the best of our knowledge, this is the first report of immortalized chicken preadipocytes and immortalization of chicken cells through restoration of telomerase activity with chTERT and chTR.

The ectopic expression of hTERT alone or in combination with oncogenes can allow various human and other mammalian somatic cells to bypass replicative senescence and attain immortality. For example, hTERT alone, or in combination with the human papillomavirus (HPV) E7 oncogene, can immortalize human preadipocytes [51–53]. hTERT has significant homology to chTERT [54], and accumulating evidence has shown that human and chicken telomere biology is similar [35]. Therefore, it is reasonable to hypothesize that hTERT may restore cellular telomerase activity and immortalize chicken cells. However, previous studies have shown that the introduction of hTERT was unable to restore chicken cellular telomerase activity and immortalize CEFs [30,31]. A recent study showed that stable transduction with hTERT could immortalize chicken feather keratinocyte stem cells, accompanied by high hTERT expression and high cellular telomerase activity [32]. These reports indicate that hTERT-mediated chicken cell immortalization may be influenced by many factors, including cell type and the expression of different components of telomerase.

TERT is the rate-limiting component of human telomerase in normal somatic cells (normal cells constitutively express the RNA component of telomerase) [19,27]; however, the rate-limiting factor of chicken telomerase in preadipocytes remains unknown. In the present study, either or both chTERT and chTR were introduced into primary chicken preadipocytes, and cell telomerase activity and cellular immortality were assessed. Our results demonstrated that transduction of chTERT alone or in combination with chTR can restore chicken telomerase activity and immortalize chicken preadipocytes, but transduction of chTR alone cannot restore chicken telomerase activity or immortalize chicken preadipocytes, suggesting that chTERT is the rate-limiting component of chicken telomerase activity in preadipocytes. Our result was consistent with a report showing that the transfection of chTR or virus TR (vTR) could not reconstitute chicken telomerase activity [35].

Although chTERT was the rate-limiting factor for chicken telomerase and transduction of chTERT alone was sufficient to immortalize chicken preadipocytes, in the present study, we demonstrated that the combination of chTERT and chTR increased telomerase activity and made the cellular immortalization easier and faster in comparison with chTERT alone (Figs 1 and 3B). The promoting effect of chTR on chicken preadipocyte immortalization is consistent with previous studies of MDV vTR [55–57]. MDV vTR is a telomerase RNA component produced by MDV that displays 88% sequence identity to chTR, exhibits supporting and promoting effects during oncogenesis, and enhances the incidence and severity of lymphoma caused by MDV [55,56]. The interaction of vTR and chTERT and the high level of vTR expression in MDV-infected lymphocytes led to an increase in telomerase activity and promoted cell immortalization [56,57]. Consistently, in the present study, ICP2 cells, in which chTERT and chTR were highly expressed, had a higher telomerase activity (Fig 3B) and a higher proliferation rate (Fig 1) in comparison with ICP1 cells. Taken together, these data suggest that chTR can facilitate chicken telomerase reactivation and cell immortalization, but chTR alone cannot immortalize chicken cells.

Several previous studies have demonstrated that hTERT alone or in combination with viral oncogenes can immortalize human primary preadipocytes [51–53]. The viral oncogenes can inactivate tumor suppressor protein p53 and retinoblastoma protein (pRB), and prevent cells from entering senescence and growth arrest during immortalization [58]. However, several viral oncogenes could disrupt the balance between cell differentiation and proliferation [59,60]. For example, transduction of SV40 large T-Ag could block the differentiation of 3T3-F442A cells into adipocytes [59], which can be explained by the fact that SV40 large T-Ag inhibits the p300/CBP coactivation function [61]. It has been shown that pRB has a specific role in stimulating adipocyte differentiation by activating C/EBPα-mediated transcription [62] and that pRB inactivation could block 3T3-L1 adipocyte differentiation [60]. Since HPV-E7 can inactivate pRB proteins [58], it is possible that, although preadipocytes immortalized by transduction of hTERT in combination with either HPV-E7 or -E6/E7 oncogenes preserved their differentiation capacity at early passage numbers [51,52], they might lose the differentiation capacity as the passage number increases. In the present study, no viral oncogene was introduced into our established cell lines, which may in part explain why our two established cell lines retained their differentiation capacity even over PD 100 (Fig 5) and were not malignantly transformed (Fig 4B).

Telomerase activity restoration may not be suitable for immortalization of all chicken cell types, such as CEFs. In the present study, we showed that chTERT alone or in combination with chTR could restore telomerase activity in chicken preadipocytes and immortalize chicken preadipocytes. However, previous studies have shown that no telomerase activity can be produced in CEFs transfected respectively with hTERT, chTERT, chTR, or both chTERT and chTR [31,35], suggesting that telomerase activity restoration varies between cell types. Interestingly, DF-1 cells, a spontaneously-occurring, immortalized telomerase-negative CEF cell line, have greater amounts of telomere DNA than primary chicken cells (CEFs) and telomerase-positive chicken cell lines (DT40) [35], suggesting that DF-1 cells use a telomerase-independent mechanism for telomere maintenance, called alternative lengthening of telomeres (ALT) [63]. ALT has been documented in human and murine tumor-derived cell lines and human immortalized cell lines [63]. A recent study confirmed that there are two telomere maintenance pathways, the telomerase and ALT pathways, which may coexist in chicken cells [44]. In the present study, chicken preadipocytes were immortalized by retroviral transduction of chicken TERT alone or in combination with TR, suggesting that the telomerase mechanism dominates in chicken preadipocytes.

Cellular DNA content variation usually implies genetic heterogeneity and reflects chromosomal aberrations [64]. It is usually caused by a higher mutation rate *in vitro* and higher cell proliferation rate. In the present study, our two established cell lines were aneuploid, and their average DNA contents were substantially different from the normal diploid DNA content (Fig 4A), suggesting that the cellular chromosome number or structure is changed in our established cell lines [65]. In fact, aneuploidy is necessary for cellular immortalization, and no permanent cell line with a strictly euploid chromosome constitution has yet been generated [66]. Immortalized cells with aneuploidy are not necessarily tumorigenic [66,67]. Consistent with this observation, in the present study, despite their aneuploidy, neither of the two established cell lines exhibited morphological features of transformation, such as the development of cell cloning foci or loss of contact inhibition in culture (Fig 2), and displayed anchorage-independent growth (Fig 4B). More importantly, our results showed that both these two cell lines retained their differentiation capacities even through PD 100 (Figs 5 and 6).

Broiler chicken obesity (excessive fat deposition) is a serious concern for the modern broiler industry that urgently needs to be addressed. Although many human and mouse immortalized preadipocyte cell lines have been established and are widely used to explore adipogenesis and obesity, no chicken preadipocyte cell lines have yet been generated, which has hindered the investigation of chicken adipogenesis and obesity. In the present study, we established two immortalized chicken preadipocyte cell lines that maintain the morphological features and differentiation characteristics of primary chicken preadipocytes, suggesting that these two cell lines could serve as *in vitro* cellular models for studying chicken adipogenesis, adipose development and obesity.

Human obesity is a serious global health concern and has been implicated in a host of diseases, including hypertension, cardiovascular diseases, type 2 diabetes and insulin resistance. There is an urgent need to understand and tackle obesity and its related diseases. Chickens are naturally hyperglycemic and insulin resistant; thus, the chicken is a potential model for studying human obesity, insulin resistance and type 2 diabetes. Our establishment of immortalized chicken preadipocyte lines may provide a potential *in vitro* model to explore the molecular mechanisms of human obesity and its related diseases.

In conclusion, our results demonstrate that chTERT, not chTR, is the rate-limiting factor for chicken telomerase restoration, and either chicken TERT alone or in combination with chicken TR can immortalize chicken preadipocytes. Our established chicken preadipocyte cell lines may serve as potential *in vitro* models for investigating molecular and cellular mechanisms underlying chicken adipogenesis and obesity.

## Supporting information

S1 Table The original data of quantitative real-time RT-PCR gene expression analysis. (XLSX)

S2 Table The original data of telomeric repeat amplification protocol (TRAP) assay. (XLSX)

S3 Table The original data of Oil Red O quantification. (XLSX)
